# Supplementary figures and images for: Systematic analyses of the MIR172 family members of Arabidopsis define their distinct roles in regulation of APETALA2 during floral transition
Source: PLoS Biol. 2021 Feb 2;19(2):e3001043. doi: 10.1371/journal.pbio.3001043 (PMC7853530; doi:10.1371/journal.pbio.3001043)

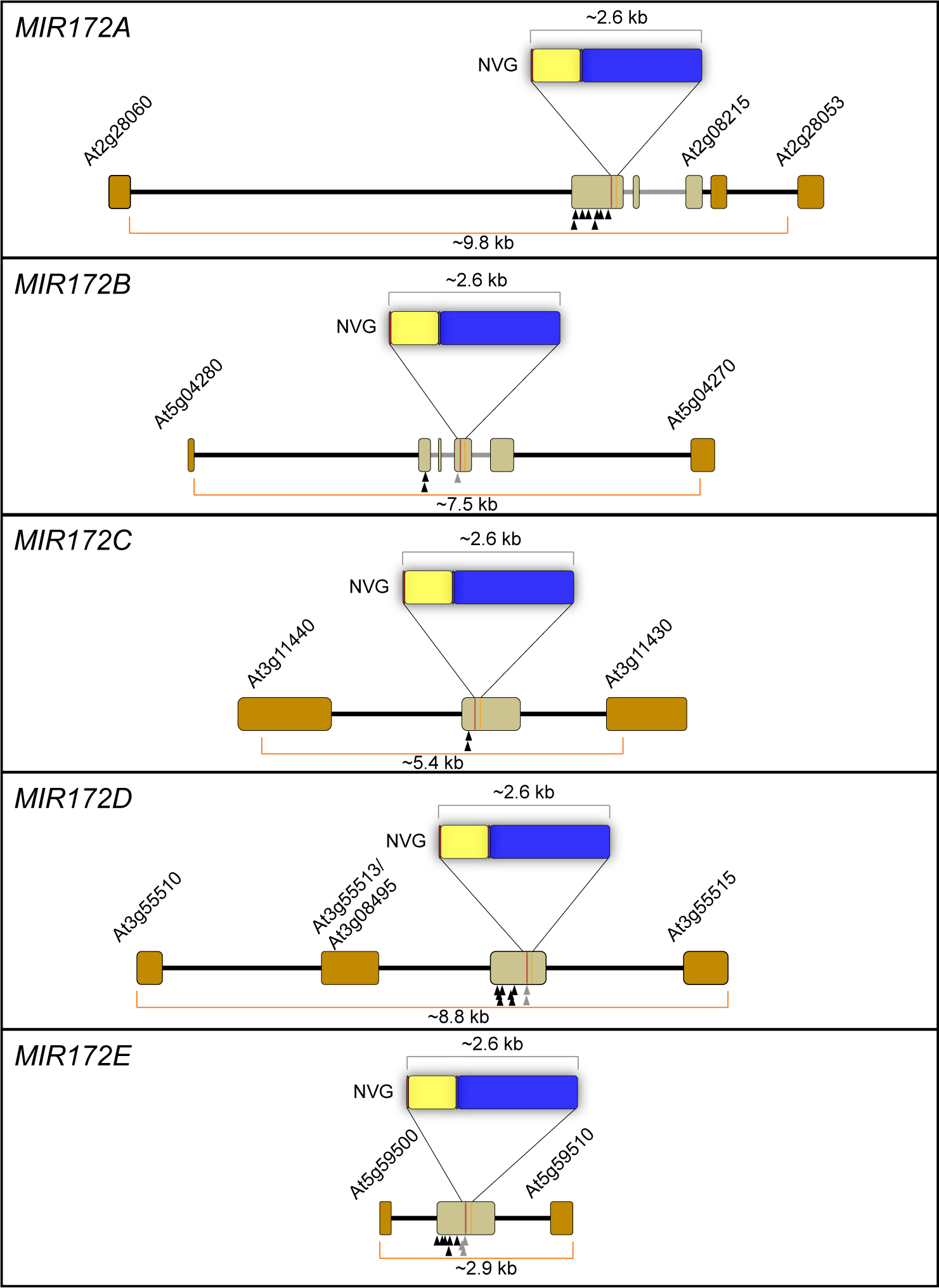

Supplement: S1 Fig — The NVG coding sequence is depicted by a short bright red box (NLS), a yellow box (Venus), and a blue box (GUS). This coding sequence was used to replace the hairpin region (dark red and orange lines) in each MIR172 gene. The beige boxes represent exons, gray lines represent introns, and black lines represent intergenic sequences including untranslated regions. Brown boxes indicate adjacent genes. At3g55513 is annotated as encoding a hypothetical protein, whereas At3g08495 and At2g08215 are annotated as expressing long noncoding RNAs, respectively, and were therefore included in the reporter constructs for MIR172A and MIR172D as they may contain regulatory elements relevant to their expression. The complete genomic structure has only been determined for MIR172A and MIR172B. Therefore, the most upstream transcription start site for MIR172C and MIR172E described in [90] were used. RNA-seq data available from Araport [91] were used to estimate which regions surrounding the hairpin coding regions of MIR172A-C are transcribed. Black arrows beneath the schematics indicate ATGs upstream of the NVG reporter, whereas gray arrows indicate ATG that were present before the MIR172 hairpin coding region that were omitted from the reporter constructs. ATG, adenine, thymine, guanine; GUS, β-glucuronidase enzyme; NVG, NLS-Venus-GUS; RNA-seq, RNA-sequencing. (TIF) [file pbio.3001043.s001.tif]

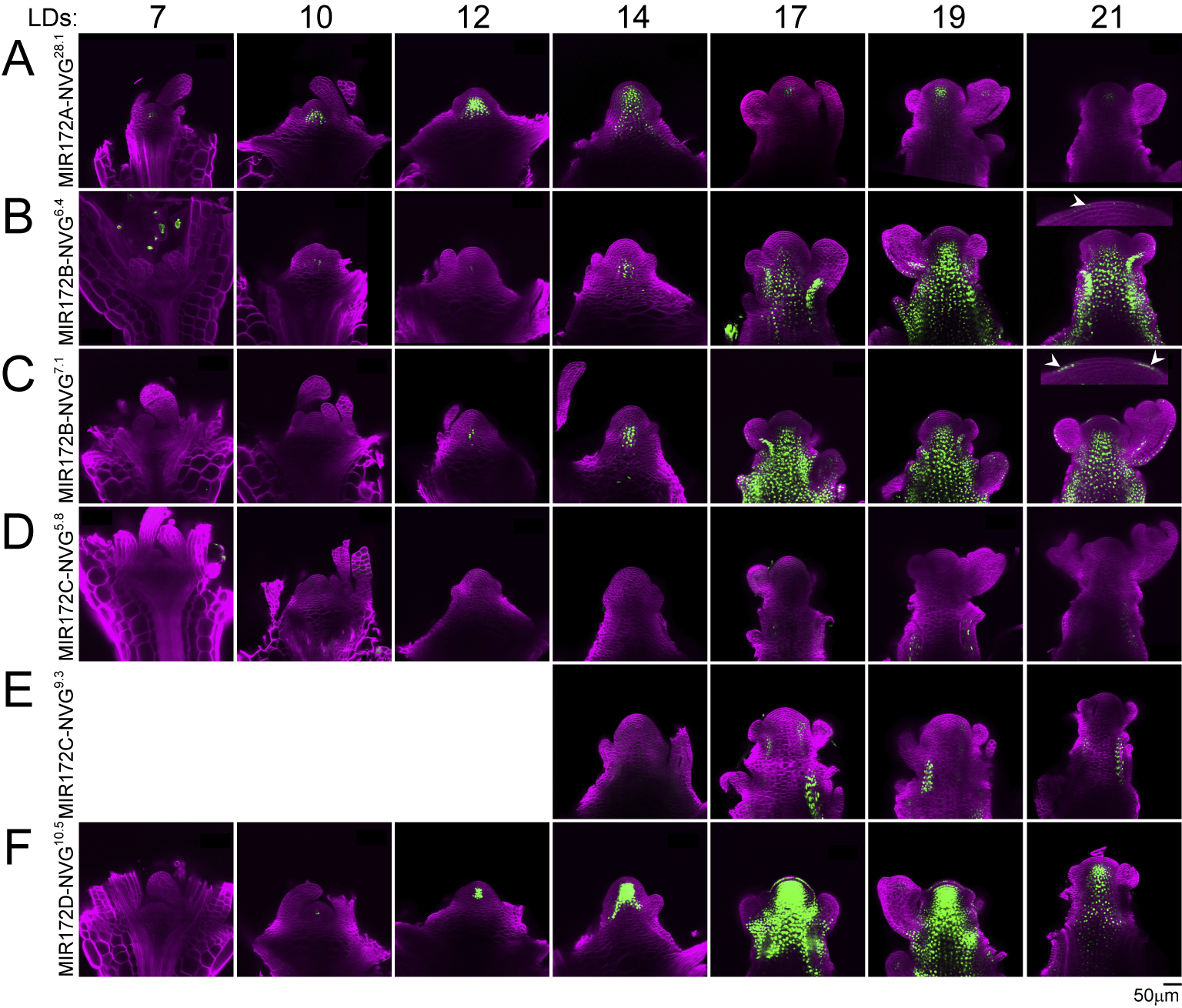

Supplement: S2 Fig — (A–F) Confocal laser scanning micrographs of the shoot apices of additional independent homozygous transgenic lines of (A) MIR172A-NVG, (B-C) MIR172B-NVG, (D, E) MIR172C-NVG, and (F) MIR172D-NVG transgenic plants grown in LD conditions and harvested at the indicated times after germination. (B, C) Note the presence of fluorescence in the L1 of each independent MIR172B-NVG line (inset, arrowheads). Fluorescence from the Venus protein is artificially colored in green, and the fluorescence from the Renaissance dye is artificially colored in magenta. LD, long-day; NVG, NLS-Venus-GUS. (TIF) [file pbio.3001043.s002.tif]

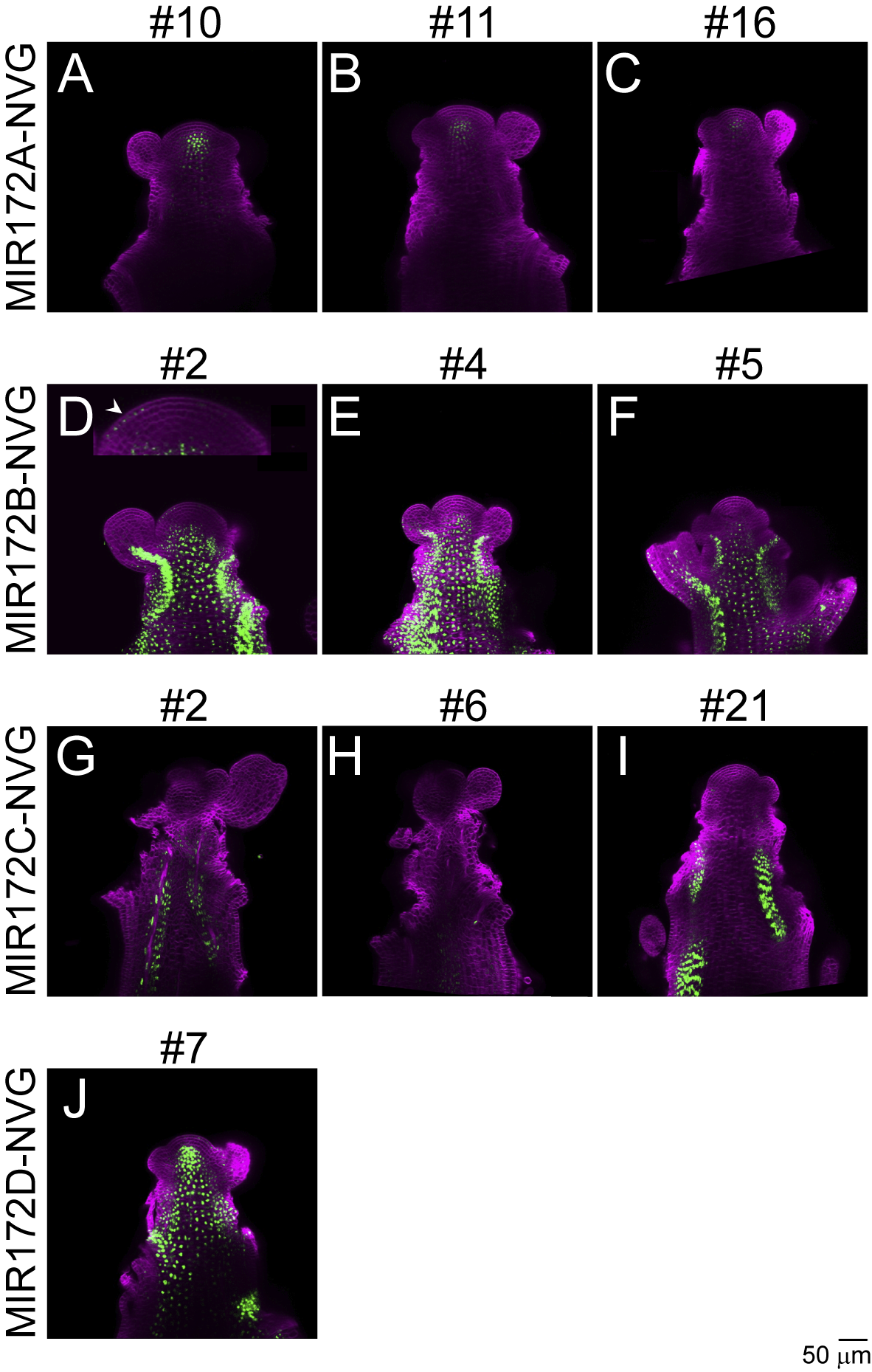

Supplement: S3 Fig — Confocal laser scanning micrographs of the shoot apices of 3 independent (A–C) MIR172A-NVG, (D–F) MIR172B-NVG, (G–I) MIR172C-NVG, and 1 independent (J) MIR172D-NVG transgenic reporter line(s). (D) Note the presence of fluorescence in the L1 of MIR172B-NVG#2 (inset, arrowheads). Fluorescence from the Venus protein is artificially colored in green, and the fluorescence from the Renaissance dye is artificially colored in magenta. LD, long-day; NVG, NLS-Venus-GUS. (TIF) [file pbio.3001043.s003.tif]

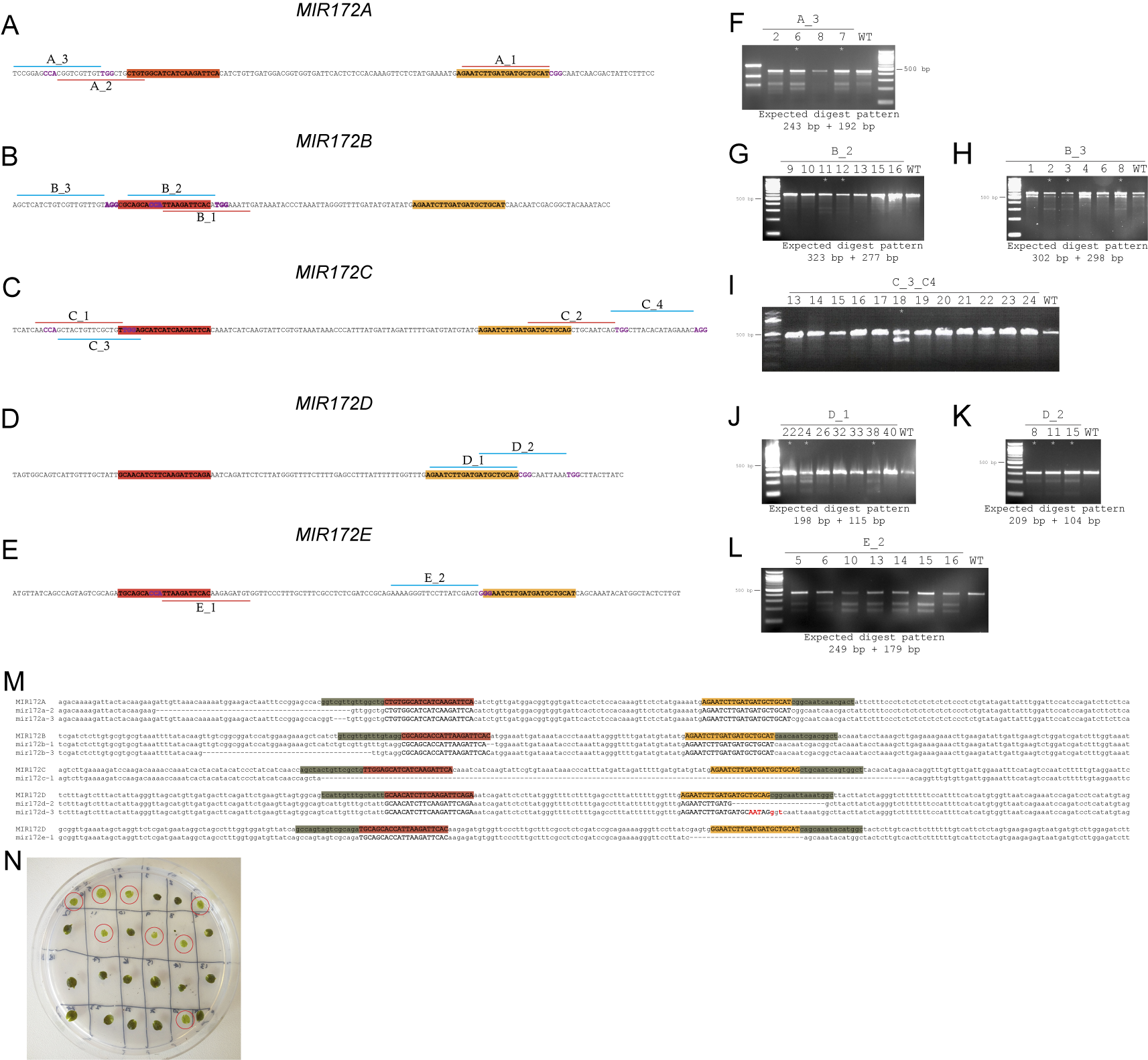

Supplement: S4 Fig — (A–E) The positions of each sgRNA are indicated (blue indicates a functional sgRNA, red indicates a non-functional sgRNA, according to the T7E1 assays). The miR172* and miR172 coding sequences are highlighted in red and orange, respectively. The PAM sequence required for functionality of the Cas-9-sgRNA complex is highlighted in purple. (F–H, J–L) Agarose gels of the indicated T7E1 assays of functional sgRNAs. DNA was derived from T1 plants transformed with the CRISPR-Cas-9 system from [53]. The expected patterns of restriction digest are indicated below the photograph, and the associated sgRNA is indicated above. (I) An agarose gel of a PCR of MIR172C using DNA derived from T1 plants harboring the CRISPR-Cas-9 system from [52], indicating the presence of a deletion (asterisk). Data underlying panels F to L are provided in S3 Data. (M) The sequences of WT MIR172A-E genes and the mutants identified by CRISPR-Cas-9. The miR172* and miR172 coding sequences are highlighted in red and orange, respectively, while the sequences required for the first processing cleavages by DCL1 are highlighted in green. Note that mir172d-3 contains several SNPs (highlighted in red) rather than a deletion. (N) An example of a PPT-resistance assay to identify plants that lack the Cas-9-containing T-DNA. Young leaves were places on an MS-agar (-sucrose) plate supplemented with 25 μg/mL PPT and incubated in a growth chamber for 3 days. The leaves derived from plants lacking the Cas-9-containing T-DNA are light green (red circles), whereas the leaves derived from plants harboring the Cas-9-containing T-DNA are dark green. Cas-9, CRISPR associated protein-9; CLN, cauline leaf number; CRISPR, clustered regularly interspaced short palindromic repeats; PAM, protospacer adjacent motif; PPT, phosphinothricin; sgRNA, single guide RNA; WT, wild-type. (TIF) [file pbio.3001043.s004.tif]

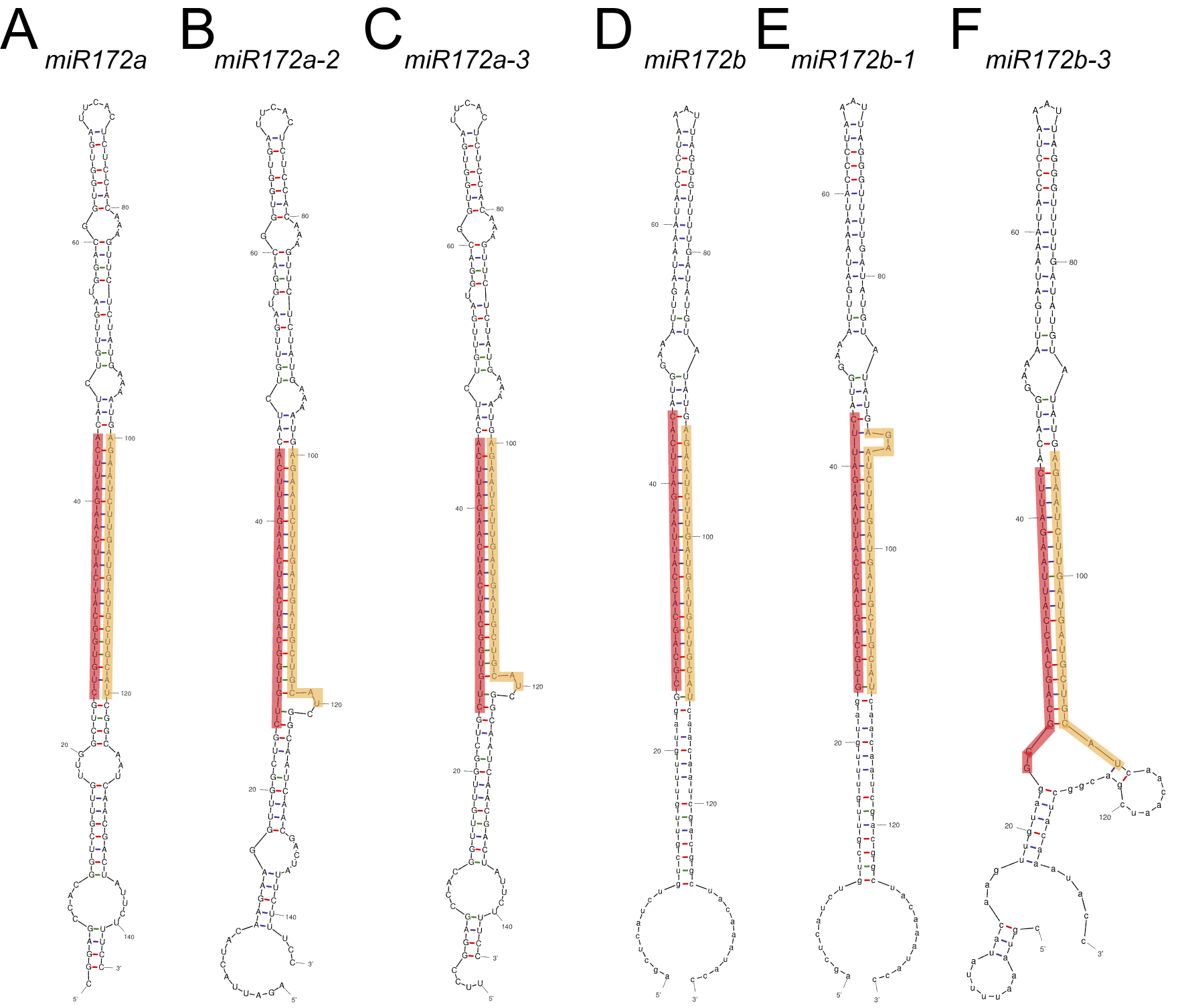

Supplement: S5 Fig — The predicted secondary structure of the pri-miRNAs derived from (A) MIR172A, (B) mir172a-1, (C) mir172a-2, (D) MIR172B, (E) mir172b-1, and (F) mir172b-3 are shown. The miR172* and miR172 coding sequences are highlighted in red and orange, respectively. miRNA, microRNA; WT, wild-type. (TIF) [file pbio.3001043.s005.tif]

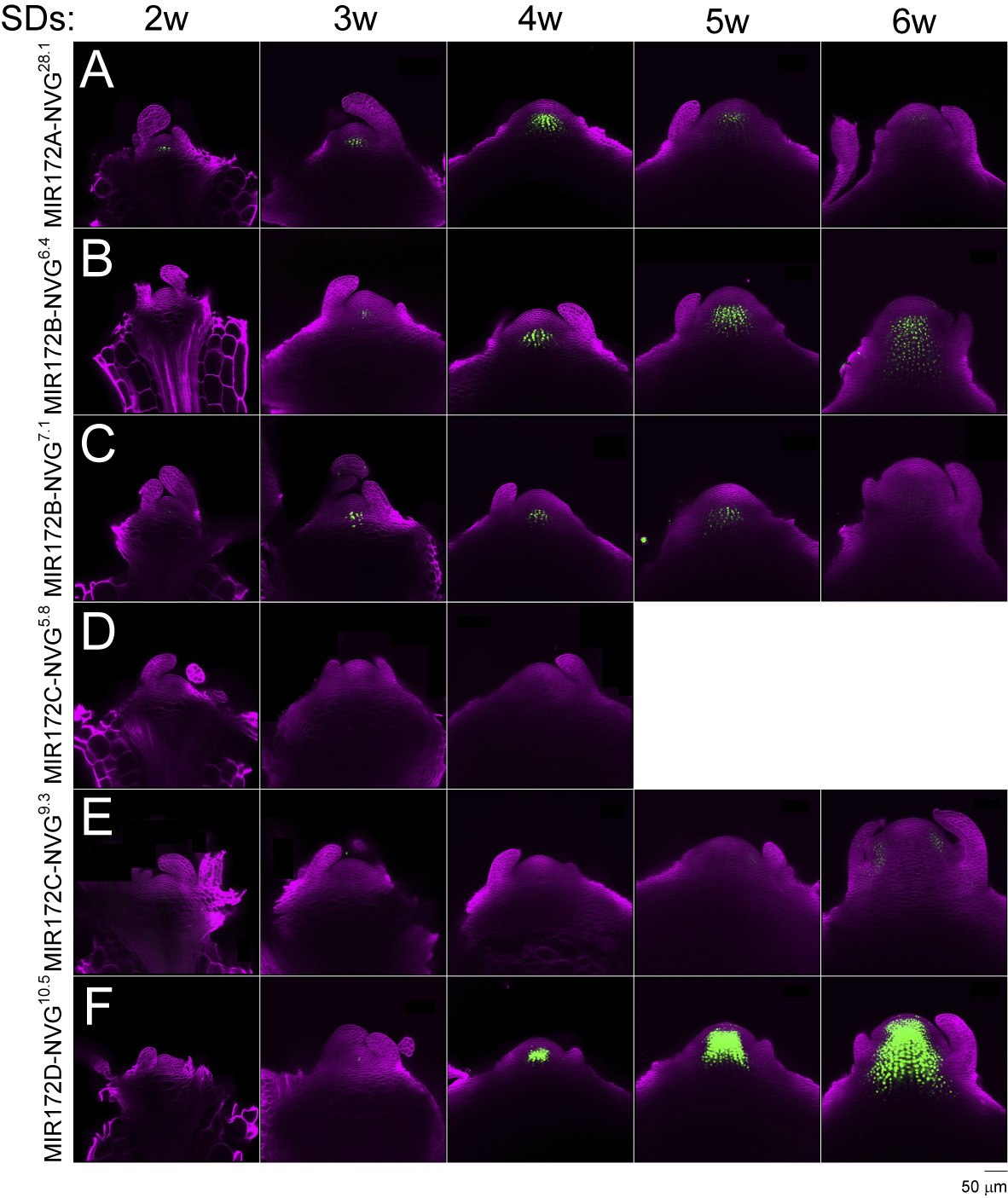

Supplement: S6 Fig — (A–F) Confocal laser scanning micrographs of the shoot apices of additional independent transgenic reporter lines of (A) MIR172A-NVG, (B, C) MIR172B-NVG, (D, E) MIR172C-NVG, and (F) MIR172D-NVG transgenic plants grown in LD conditions and harvested at the indicated times after germination. Fluorescence from the Venus protein is artificially colored in green, and the fluorescence from the Renaissance dye is artificially colored in magenta. LD, long-day; NVG, NLS-Venus-GUS; SD, short-day. (TIF) [file pbio.3001043.s006.tif]

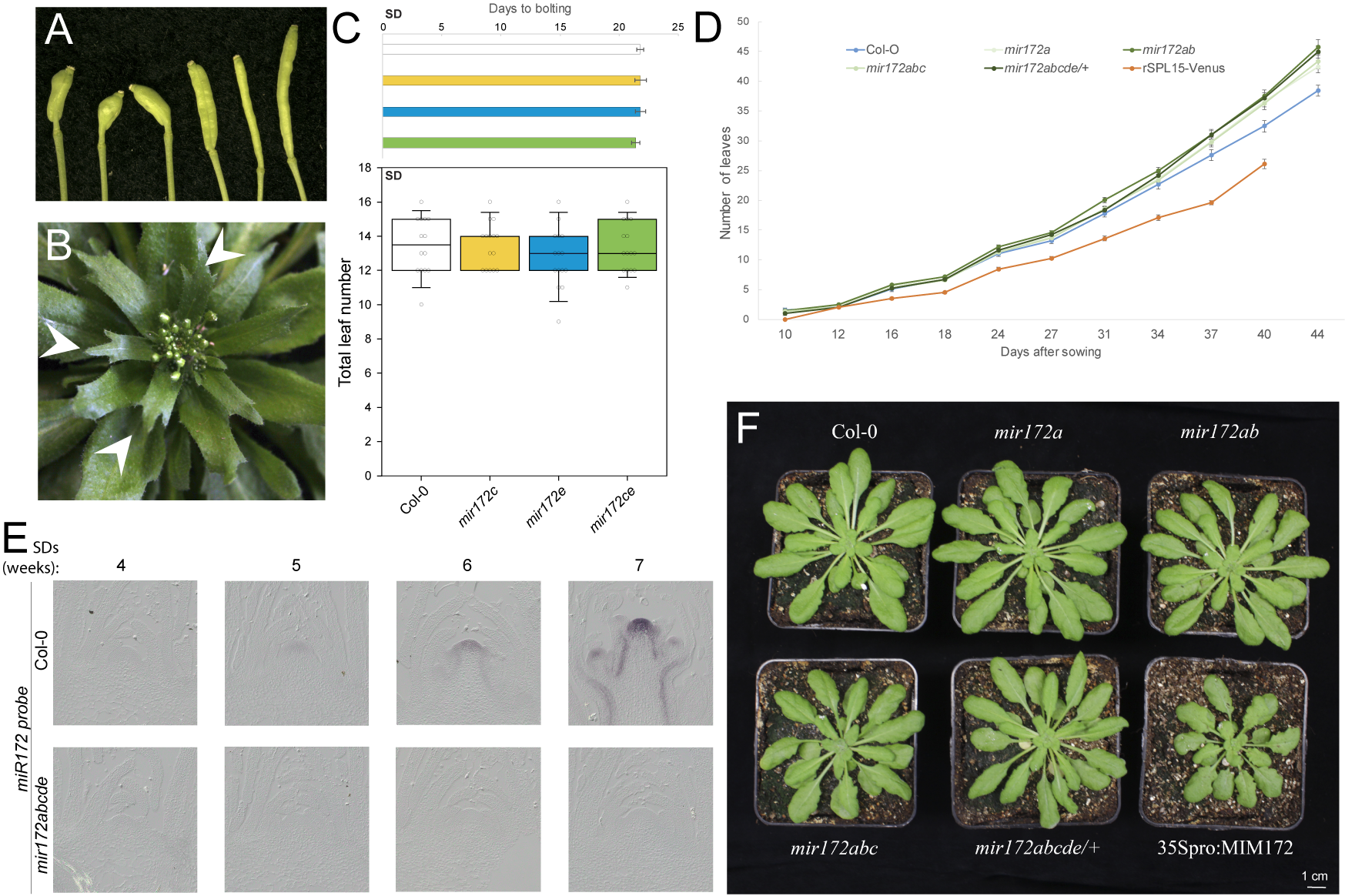

Supplement: S7 Fig — (A) A photograph of mir172a-2 b-3 c-1 d-3 siliques grown in LD conditions from a single plant. The ages of the siliques are in order from left (early arising) to right (late arising). Note that the phenotype is most severe in the first flowers/siliques to arise. (B) A photograph of mir172a-2 b-3 d-2 inflorescence. Note the extremely serrated cauline leaves (arrowheads). (C) Graphs depicting the TLN and DTB of Col-0, mir172c-1, mir172e-1, and mir172c-1 e-1 grown under LD conditions. (D) The number of leaves present on SD-grown plants that were larger than 0.25 cm on each indicated day. Error bars indicate SEM. (N = 12–16). Statistical comparisons relative to Col-0 at 40d: mir172a; Δ4.18 RLN; p = 0.001), (mir172ab; Δ5.03 RLN; p < 10–5), (mir172abc; Δ3.75 RLN; p = 0.003), (mir172abcd(e/+); Δ4.69 RLN; p = 0.0004). The Venus-rSPL15 genotype was used here as a control. Data underlying panel D are provided in S2 Data. (E) Micrographs of RNA in situ hybridizations using a commercial probe designed to recognize miR172 of Col-0 (upper panels) and mir172a-2 b-3 c-1 d-3 e-1 (lower panels) apices grown under SD conditions for the indicated times. (F) A representative photograph of the morphologies of plants of the same age grown under SD conditions. The genotypes used in each experiment were Col-0, mir172a-2, mir172a-2 b-3, mir172a-2 b-3 c-1, mir172a-2 b-3 c-1 d-3 e-1/+, 35Spro:MIM172, and Venus-rSPL15. DTB, days to bolting; LD, long-day; SEM, standard error of the mean; SD, short-day; TLN, total leaf number. (TIF) [file pbio.3001043.s007.tif]

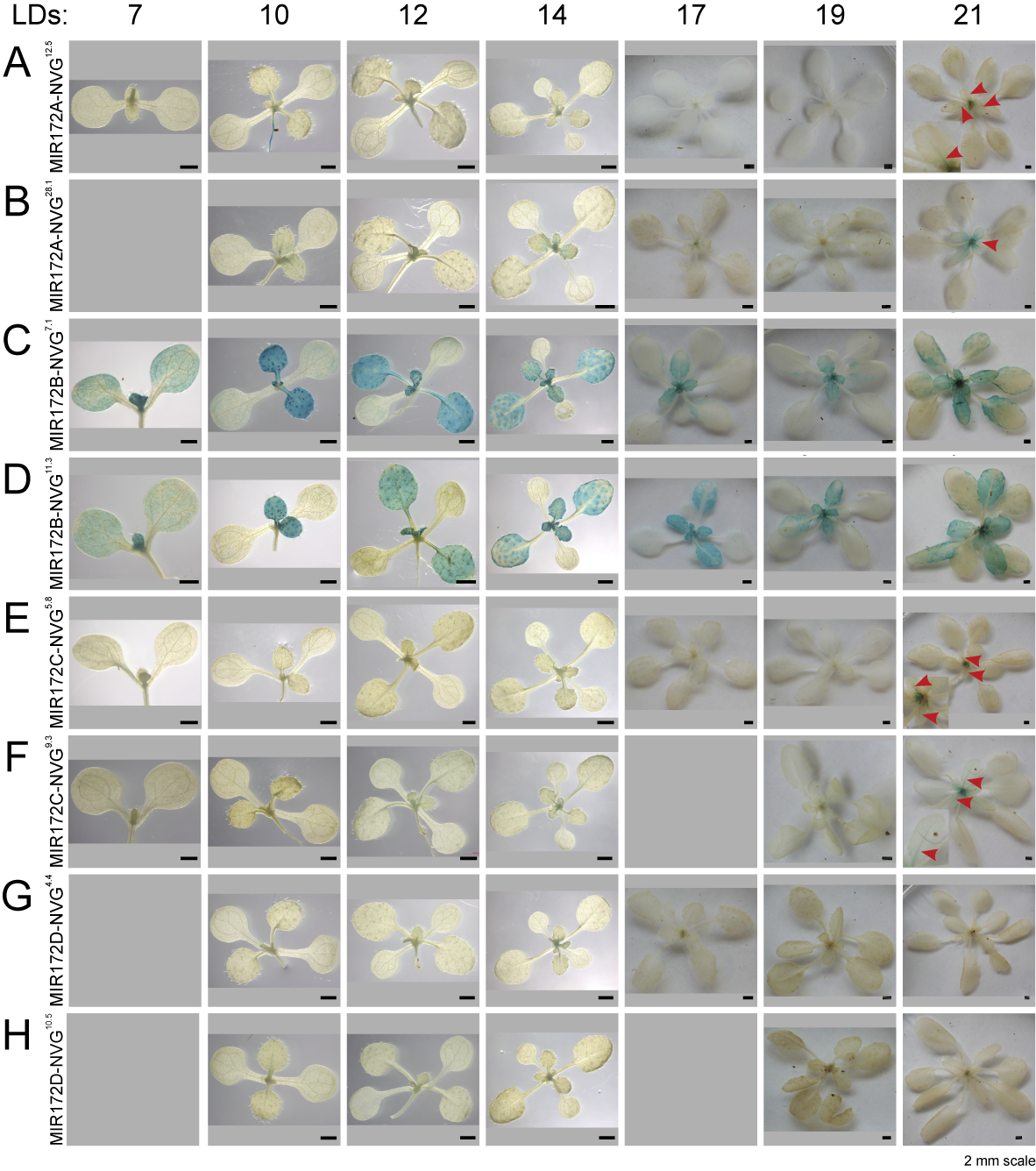

Supplement: S8 Fig — Photographs of 2h GUS staining of independent homozygous transformants for (A, B) MIR172A-NVG, (C, D) MIR172B-NVG, (E, F) MIR172C-NVG, and (G, H) MIR172D-NVG grown under LD conditions and harvested at the indicated times. Red arrowheads indicate the presence of weak GUS staining. GUS, β-glucuronidase enzyme; LD, long-day; NVG, NLS-Venus-GUS. (TIF) [file pbio.3001043.s008.tif]

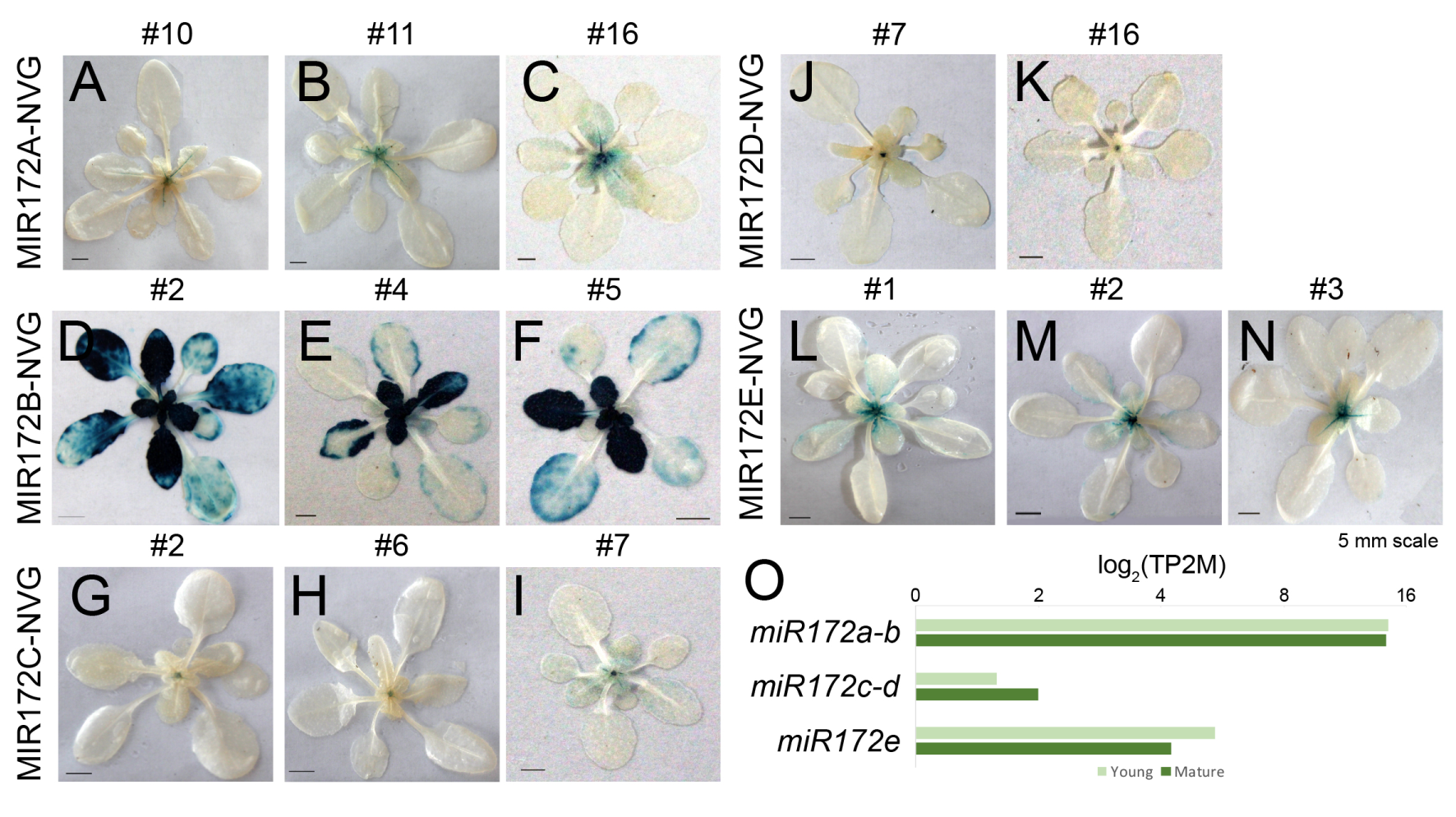

Supplement: S9 Fig — Photographs of overnight GUS staining of 3 independent second generation transformants for (A–C) MIR172A-NVG, (D, E) MIR172B-NVG, (G, I) MIR172C-NVG, (L–N) MIR172D-NVG, and (J, K) 2 independent second generation transformants grown under LD conditions and harvested 21 d after germination. (O) A graph indicating the log2 TP2M for each miR172 isoform in the leaves of A. thaliana (data from [56]). Note that the isoform arising from MIR172A and MIR172B, and MIR172C and MIR172D, are identical, respectively. Data underlying panel O are provided in S1 Data. GUS, β-glucuronidase enzyme; LD, long-day; NVG, NLS-Venus-GUS; TP2M, transcripts per 2 million. (TIF) [file pbio.3001043.s009.tif]

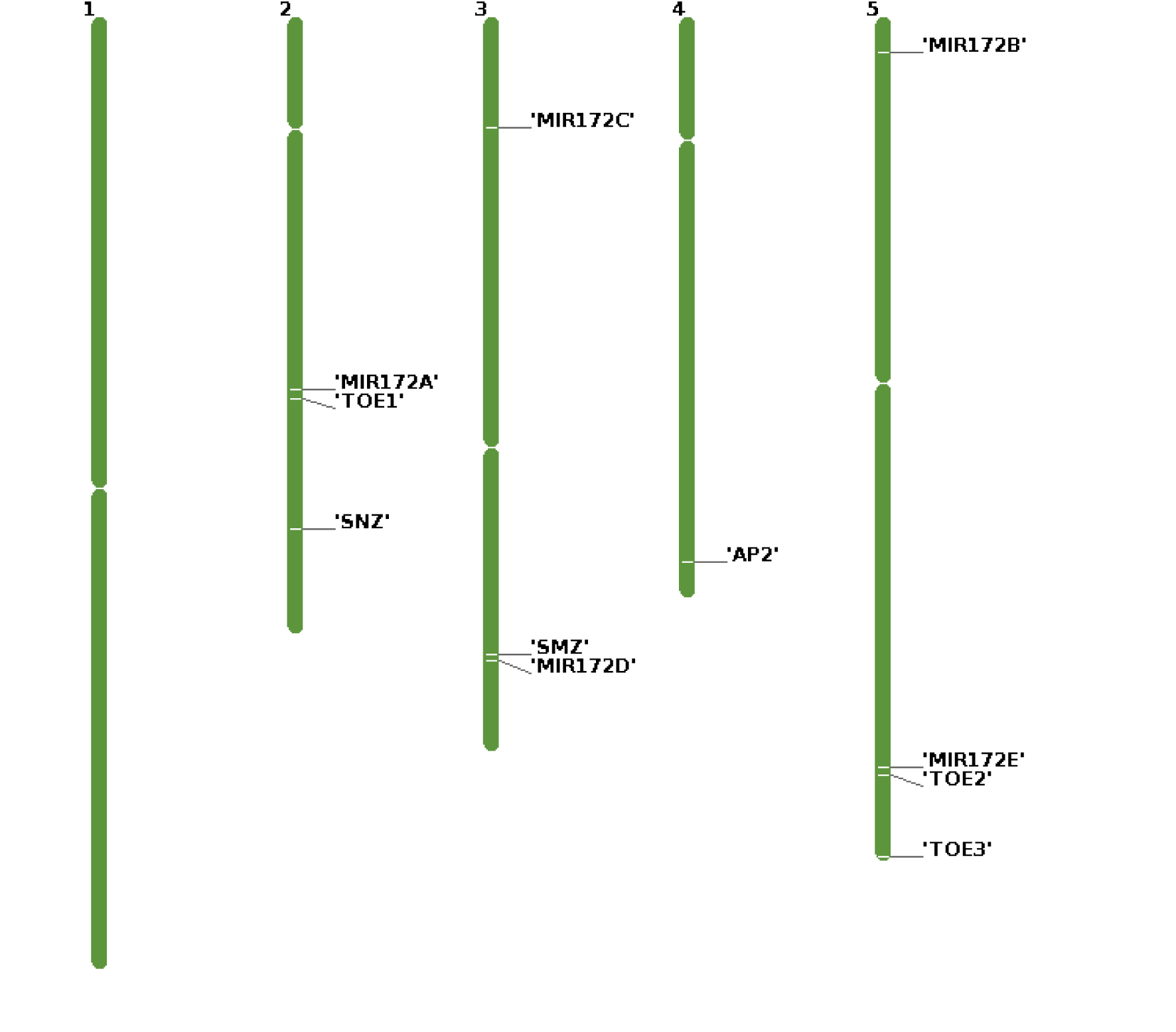

Supplement: S10 Fig — Visualization of the chromosomal locations of each MIR172, AP2-LIKE, SPL15, and FUL genes in A. thaliana. Map was created with the Chromosome Map Tool provided by TAIR (www.arabidopsis.org). AP2-LIKE, APETALA2-LIKE; FUL, FRUITFULL; SPL, SQUAMOSA PROMOTER BINDING PROTEIN-LIKE; TAIR, The Arabidopsis Information Resource. (TIF) [file pbio.3001043.s010.tif]

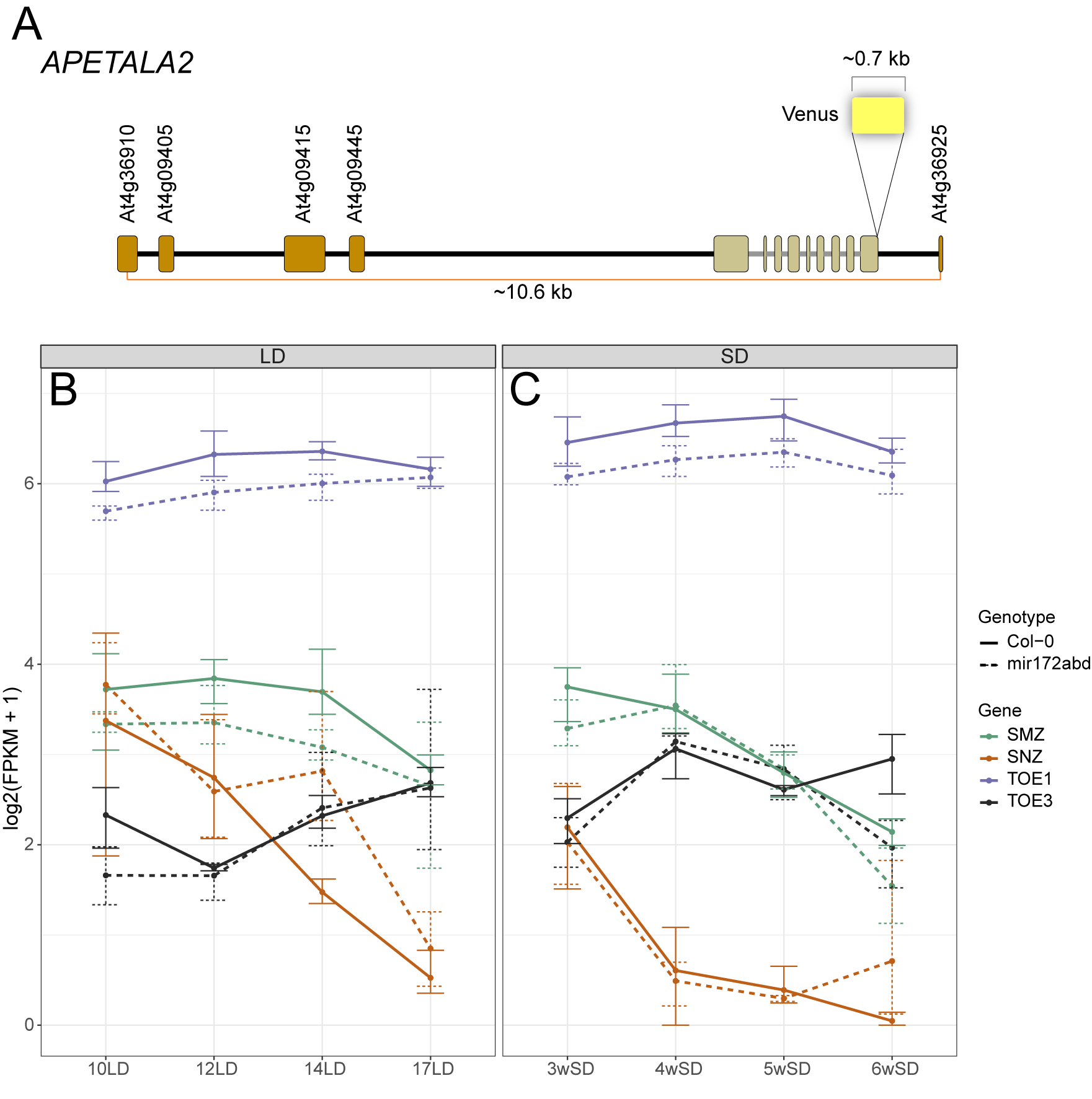

Supplement: S11 Fig — (A) The Venus coding sequence is depicted by a yellow box. This coding sequence was fused in frame to the final exon of AP2 by removing the stop codon of AP2. The beige boxes represent exons, gray lines represent introns, and black lines represent intergenic sequences including untranslated regions. Brown boxes indicate adjacent genes. At4g09445/At4g09415 are annotated as long noncoding RNAs, and At4g09415 is annotated as “other RNA” and were therefore included in the reporter construct as they may contain regulatory elements important for AP2 expression. (B, C) Graphs depicting RNA-seq-derived data of the mRNA levels in apices of SMZ, SNZ, TOE1, TOE3, and AP1 in Col-0 and miR172abd at the indicated time points after germination in (B) LDs and (C) SDs. Error bars represent the standard deviation of 3 biological replicates. Data underlying panels (B) and (C) are provided in S1 Data. AP2, APETALA2; AP2-LIKE, APETALA2-LIKE; LD, long-day; RNA-seq, RNA-sequencing; SD, short-day. (TIF) [file pbio.3001043.s011.tif]

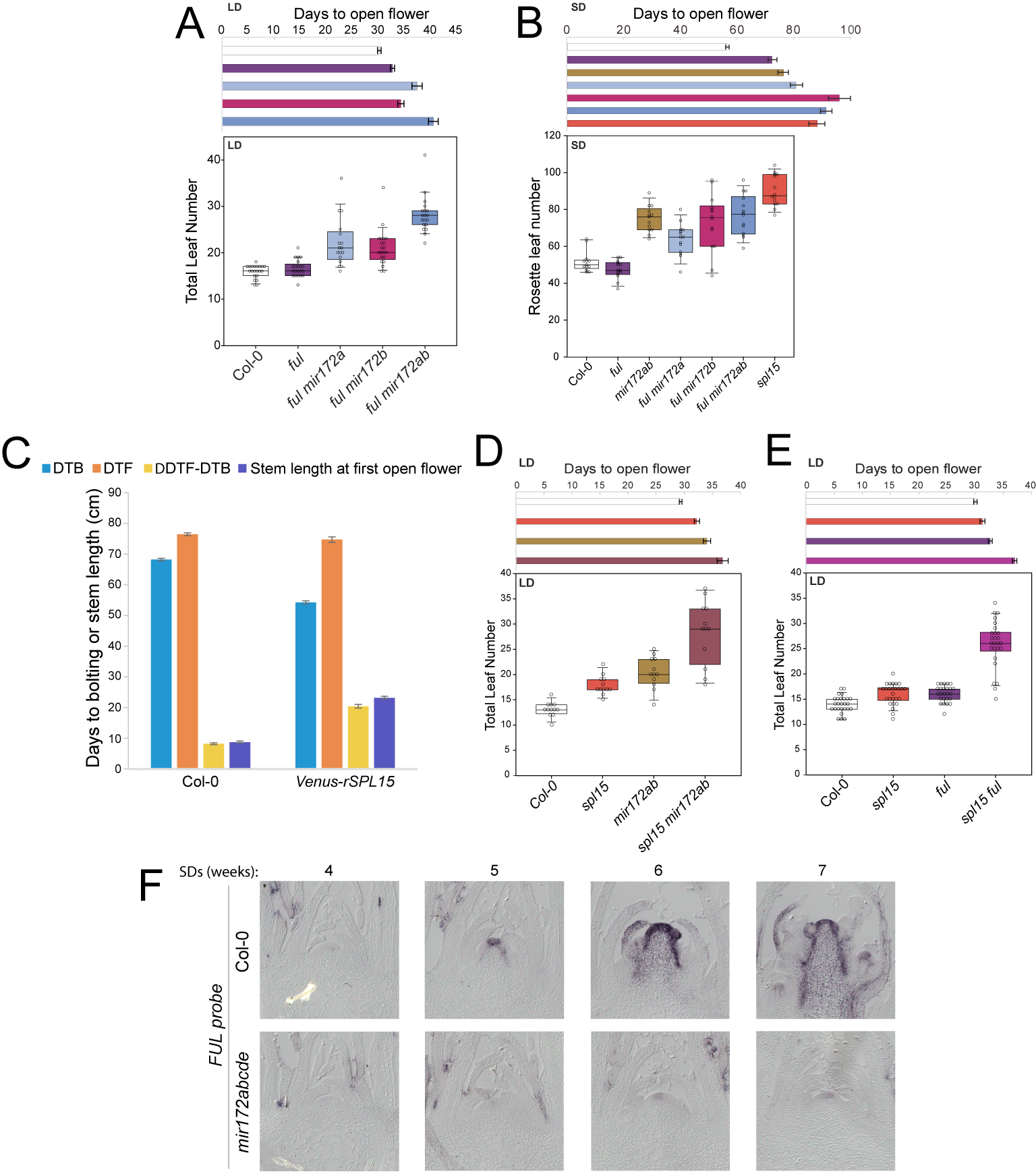

Supplement: S12 Fig — (A, B) Graphs depicting DTF and TLN between the indicated combinations of ful-2 and mir172 mutants grown in (A) long and (B) SD conditions. (C) A bar chart depicting the DTB, DTF, the duration between DTB and DTF (ΔDTF-DTB), and the stem length at flowering. (D, E) Graphs depicting the days to open flower and TLN of combinations between spl15-1, ful-2, and mir172-a2 b-3 in LD conditions. Data underlying panels A to E are provided in S2 Data. (F) Micrographs of RNA in situ hybridizations using a probe designed to recognize FUL of Col-0 (upper panels) and mir172a-2 b-3 c-1 d-3 e-1 (lower panels) apices grown under SD conditions for the indicated times. DTB, days to bolting; DTF, days to the first flower opening; FUL, FRUITFULL; SD, short-day; TLN, total leaf number. (TIF) [file pbio.3001043.s012.tif]

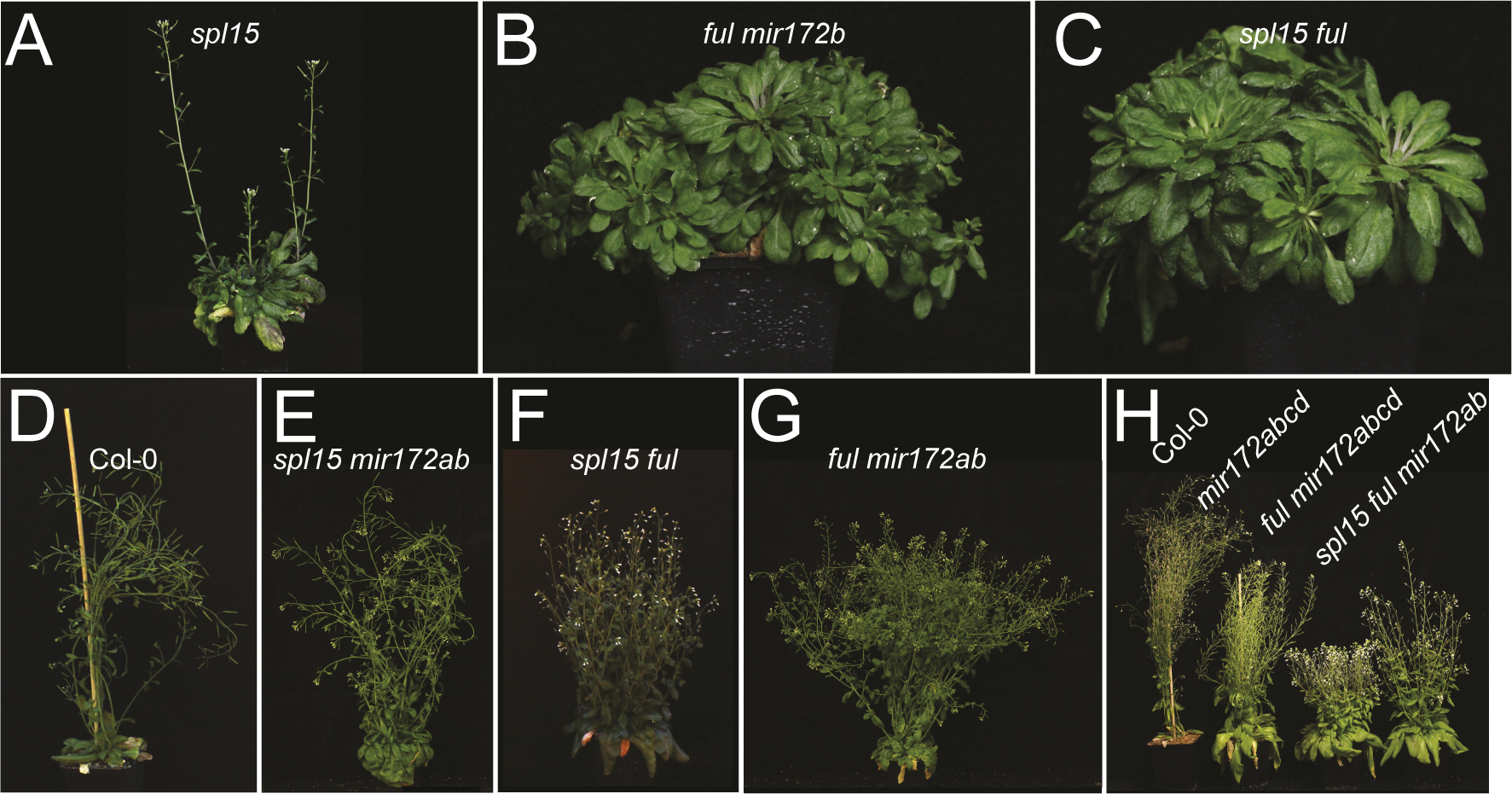

Supplement: S13 Fig — Flowering architecture of indicated combinations between spl15-1, ful-2, and mir172 in (A–C) SDs and (D–H) LDs. LD, long-day; SD, short-day. (TIF) [file pbio.3001043.s013.tif]

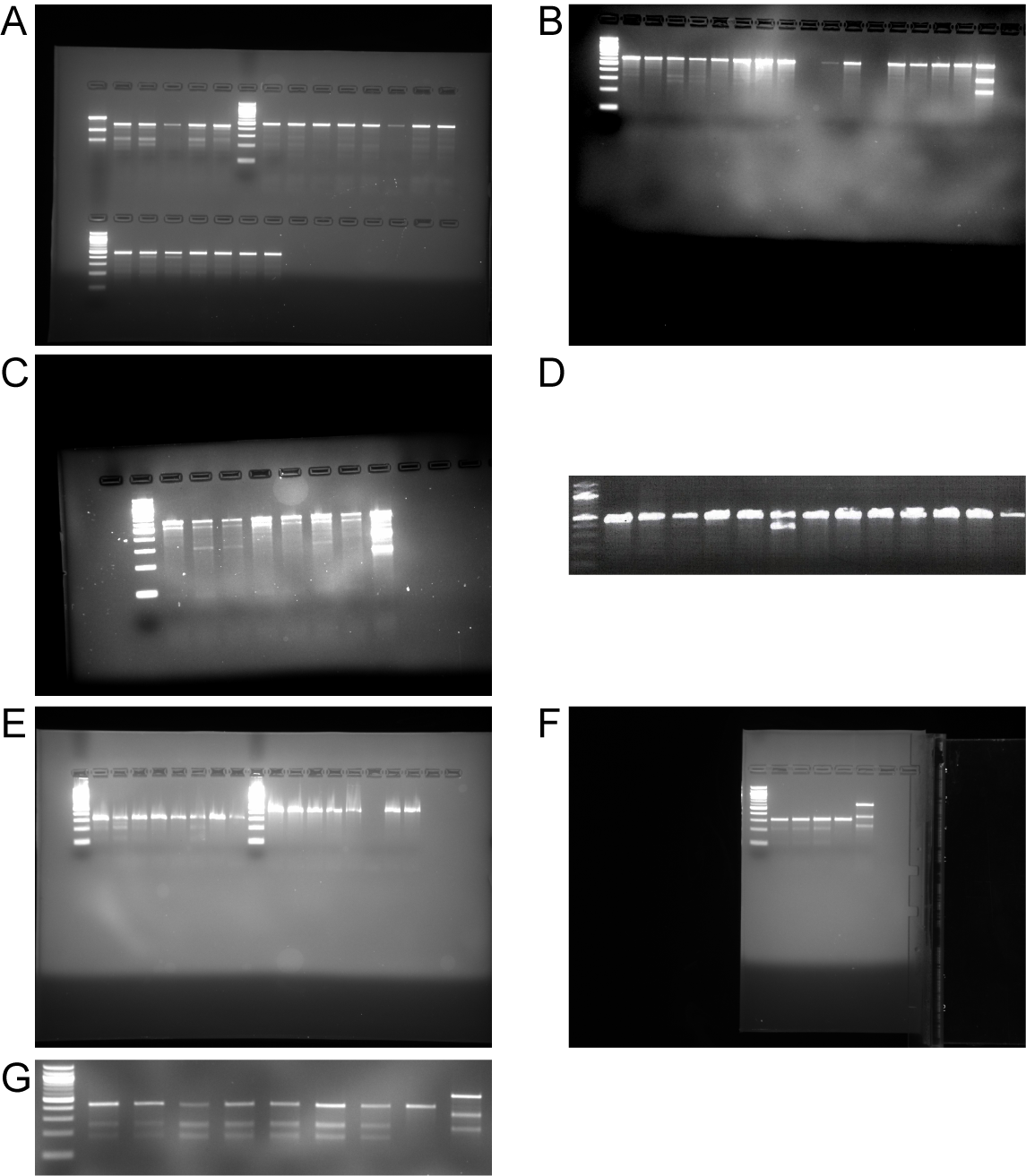

Supplement: S3 Data — (TIF) [file pbio.3001043.s019.tif]
