# Supplementary material for: Systematic analyses of the MIR172 family members of Arabidopsis define their distinct roles in regulation of APETALA2 during floral transition
Source: PLoS Biol. 2021 Feb 2;19(2):e3001043. doi: 10.1371/journal.pbio.3001043 (PMC7853530; doi:10.1371/journal.pbio.3001043)
Supplement: Attachment — Submitted filename: Cover letter_response.docx [file pbio.3001043.s020.docx]

Dear Dr Alvarez-Garcia,

Thank you for your Email of 23^rd^ September 2020 enclosing the decision letter and reviews of our manuscript "Analysis of the *MIR172* family defines transcriptional and post-transcriptional mechanisms that coordinately regulate APETALA2 to control floral transition of Arabidopsis" (PBIOLOGY-D-20-01959R1). We were very pleased that we were invited to submit a revised version of the manuscript that takes account of the reviewers’ comments, and that the reviewers found our manuscript interesting. We have addressed each of the reviewers’ comments below. Their text is in italics, and our responses follow.

**Reviewer 1**

The reviewer summarises with the comment*: “This is a well-written and organized paper. The genetic analysis is sophisticated and related genetic data including CRISPR-induced mutants of each miR172 are exciting and convincing. I have a few minor comments.”* We respond to each comment below.

1. *In Fig4, the analysis of the each MIR172 promoter activity suggests that MIR172B could be the main contributor for the miR172 pool, which was also supported by the measurement of mature miR172 levels in miR172b and miR172 quintuple mutant. How is the accumulation level of mature miR172 in other miR172 single mutants? Since the level of miRNA can be determined not only at the transcriptional level but also at the processing step, I would be nice to clarify it.*

The reviewer is right to point out that in Figure 4D, we tested by RT-qPCR mature mir172 levels in *mir172b* and *mir172abcde* mutants. We found that in the quintuple mutant, mir172 levels were less than 1% of those in wild-type plants, whereas in *mir172b* mutants they were 27-50% of wild-type, as described on page 15. We did not measure the individual contribution of the other 4 genes, and do not have these data to include. However, that the isoform of miR172 encoded by *MIR172b* and *MIR172a* is the major contributor to the mir172 pool in leaves was also supported by our analysis of the miRNA sequencing data of Thatcher et al (2015). In these data, we found that the isoform encoded by *MIR172A*/*B* was approximately three orders of magnitude higher than those encoded by *MIR172C*/*D* or MIR172E, as mentioned on page 15 (Figure S9). We have edited our statement on page 15 to read that “*MIR172B* makes a major contribution” to miR172 levels in the leaves, rather than as we previously stated that “mir172 in the leaves was largely derived from *MIR172B*”. We have also inserted a statement for clarity on page 15 that “we did not test miR172 levels in the other single mutants”.

2. *In Fig 6A, please determine the AP2 expression level (may include other AP2-like genes) in the shoot apex in ful, mir172abcd and ful mir172abcd in a similar way of Fig 1A-F. Although it is known that FUL and miR172 repress AP2-like genes, it still needs to show that the additively delayed flowering in ful mir172abcd mutants is indeed due to the mis-regulation of AP2-like genes in the shoot apex to support the author's conclusion.*

We agree with the reviewer that measuring the mRNA level of *AP2* and *AP2-L* genes in the apices of *ful mir172abcd* compared to *mir172abcd* is a logical experiment to support the interpretations of the genetic experiments. However, we do not have these data, and this experiment would take many weeks to complete, as the *ful mir172abcd* quintuple mutant is extremely late flowering. In inflorescence meristems 3 weeks after flowering, Balanza et al (2018) detected a two-fold increase in *AP2* mRNA in *ful* mutants compared to wild-type, supporting our interpretation. We have provided in response to reviewer 3 point 2 an RNAseq experiment to measure *AP2* and *AP2-L* transcripts in the mir172abd mutant, and this implies that feed-back regulation as well as post-transcriptional regulation of *AP2* and *AP2-L* genes by miR172 play important roles in determining mRNA levels. Therefore, measuring *AP2-L* transcripts in the *ful mir172abcd* is likely to be a complex and time-consuming experiment, requiring a time course and the appropriate controls. In response to the reviewer’s request for these data, we have inserted an additional comment in the Results on page 20 citing again the work of Balanza et al (2018) and emphasizing throughout that our interpretation is based on the genetic analysis (page 20: “This genetic redundancy probably involves the suppression of *AP2-LIKE* activity through transcriptional and post-transcriptional means, as was observed in mature inflorescences [47]”). We also added a sentence in the Discussion on page 26 where we discuss the synergistic interaction between *ful* and *mir172abcd* in flowering time stating that it still needs to be tested at the molecular level that this is due to dual regulation of *AP2* and *AP2-L* genes (Page 26: Taken together, this genetic analysis indicates that FUL and miR172 control *AP2-LIKE* gene activity at both the transcriptional and post-transcriptional levels to regulate the floral transition, although this remains to be tested at the molecular level.)

3. *Ref.33 is not the right reference. The authors probably mean this paper: POWERDRESS and Diversified Expression of the MIR172 Gene Family Bolster the Floral Stem Cell Network.*

Yes, the wrong reference was inserted here. We have changed reference 33 to Yumul et al (2013) throughout the text.

**Reviewer 2**

This reviewer mentions that “*This study really clarifies the role of miR172 in flowering time and its role at the shoot apex*” and that “*I think this is a strong contribution worth publishing*”. Two points are raised:

1. “*the first paragraph of the intro about feedback loops between miRNA and TF; this does not really appear to relate to plant biology, but rather animals, so seems a bit misleading*”.
   We agree with the reviewer that the sentence in the first paragraph describing robustness and feedback loops refers to examples from animal systems, and we have now edited that sentence to make this clear. We would like to retain this point, because such functions are also likely to occur in plant systems, although they have not yet been defined in detail. The other points in the first paragraph already distinguish examples from plants and animals.
2. *Typo, missing sentence? - Furthermore,*

We deleted this typo on page 13.

**Reviewer 3**

The reviewer comments that “*the results will be of wide interest to the scientific community*”, but has “*some comments about the manuscript*”.

1. *Figure 2E, I think there is something odd with the Total Leaf Number of transgenic plants overexpressing wild-type MIR172 precursors as they usually result in plants with much lower number of leaves. That wt MIR172 precursors have little effect, makes it difficult to discard a partial activity of the mir172 mutants. I think the authors should determine the mature miR172 levels in the CRISPR-Cas9-induced mutants to additionally validate the penetrance of the employed strategy*.

In this overexpression strategy, we used the *UBQ10* promoter rather than *35S* because we wanted equal distribution of miR172 expression across plant tissues and *35S* is not homogenous in its expression, particularly in the meristematic regions, however the *35S* promoter has the advantage of being stronger than the *UBQ10*. This may explain the relatively weaker phenotype the reviewer mentions. However, we did measure the level of mature mir172 in the *mir172abcde* quintuple mutant as suggested by the reviewer. Using RT-qPCR and *in situ* hybridization mir172 was almost undetectable at 1% or less of wild-type levels, supporting the strength of these mutations (Figure 4D, E). We have edited the concluding sentence of the section describing the transgenic approach so that it now mentions that the conclusions of the transgenic approach are further supported by the expression analysis in higher order mutants that is presented later. (page 11: These data indicated that the *mir172* mutant alleles were strongly impaired in function and may be null alleles, and this was further supported by testing mir172 levels in higher order mutants (see below)).

2. *Overall, I think it is important to improve the description of the expression of miR172-target genes in a mir172 mutant background. For example, can the levels of AP2-like transcription factors be determined by RT-qPCR in wt and mir172 mutants (using plants in the same developmental phase)? In Figure 5, the pattern of AP2-Venus (protein) reporter (Fig 5A) does not match the pattern of AP2 RNA (Fig 5B), even in the mir172 mutant. For example, the AP2-Venus protein seems to be expressed in the meristem, while the AP2 RNA is present in other tissues much stronger than in the meristem. Furthermore, AP2-venus has a peak at 14d in wt, while it has a peak at 19d in mir172abd mutants. Wouldn't it be expected that the miR172-target AP2 accumulates at higher levels at earlier stages in the mir172 mutant compared to wt? Please revise and discuss these data.*

We agree with the reviewer that it will be important to use our genetic material to dissect the role of miR172 in regulating AP2 and AP2-L genes during floral transition. However, this is complex, because miR172 can regulate AP2 and AP2-L transcript levels by cleavage and regulate translation of the mRNAs, while AP2 and AP2-L proteins are proposed to feedback to repress their own transcription. To reduce the complexity of Figure 5A, we have removed the two early time points, where the AP2-VENUS signal was weak. The time course now starts at 14 days, where AP2-VENUS is clearly detectable in Col, and then disappears at 17 days as the meristem becomes an inflorescence meristem. In the *mir172abd* plant, which flowers later, AP2-VENUS signal is not present at 14 days, and appears strongly later. As mentioned by the reviewer, we initially expected that AP2-VENUS would be expressed at the same time points in mir172abd as in Col, so from 14 days, and then persist longer due to the loss of mir172. However, we assume that the lack of a signal at 14 days is due to cross regulation by other AP2-L proteins that are present at higher levels in the mutant. In this regard, we note that the *ap2* mutation only partially suppresses the late flowering of the mir172abd triple mutant (Figure 5). We have extended the description of these data in the Results on pages 17-18, and in the Discussion on page 26-27. Furthermore, to address this important issue, we have provided a new RNAseq experiment comparing the transcriptome at the shoot apices of the late-flowering *mir172abd* triple mutant with wild-type plants through time courses in LDs and SDs. We have extracted the mRNA levels for *AP2* and the 5 *AP2-L* genes, and provide the data for *AP2* and *TOE2* in Figure 5C, D and for the other four genes in Supplementary Figure 11. This experiment clearly shows that the mRNA of *TOE2* is increased strongly in *mir172abd* triple mutants, particularly under SDs. This is consistent with the previous reports of Schwab et al (2005) that *TOE2* mRNA was strongly reduced in plants overexpressing *MIR172A*. However, we did not find strong differences in the mRNA level of *AP2* or the remaining four *AP2-L* genes. This is also consistent with the observation of Schwab et al (2005) in *MIR172A* overexpressor plants. Nevertheless, the genetic experiment we provide indicates that AP2 does play a significant role in repressing flowering of the *mir172abd* triple mutant, and we do detect expression of *AP2::AP2-VENUS* at later time points in *mir172abd*. We have now altered the results section in pages 17-18 to mention the RNA-seq approach and to state the effect of the *mir172abd* triple mutant on *TOE2* mRNA. In the Discussion on page 26 we mention the previous evidence for feedback regulation and that the complex pattern detected for AP2-VENUS in *mir172abd* is likely due to the combination of different layers of regulation involving alteration in cleavage of the mRNA, translational repression, altered feedback regulation and cross-regulation by other AP2-L proteins.

3. *Figure 4E. Please, indicate whether there are differences in the anatomy and/or developmental phase of the plants. This also applies to Figure 5, as differences in gene expression can be explained by differences in the plant developmental phase and/or anatomy rather than a consequence of miR172 activity.*

There are differences in the anatomy and developmental phase of wild-type and *mir172* higher-order mutants, which is illustrated in the meristem sections shown in Figure 4E or Figure 5. However, to minimize these complications, we show a time course in Figure 4E. The meristem section of 28-day old *mir172abcde* plants shows an apex with floral buds, and is roughly equivalent to the 22-day old apices of wild-type plants. The miR172 signal is clearly detected before and after 22 days in WT, but is not detected in the *mir172abcde* mutant, even at 28 days. So, we are confident that the developmental stage of the material does not affect our conclusion in this experiment. Similar issues apply in Figure 5 where time courses are shown.

4. *The reporters for MIRNA expression were designed by replacing the precursor sequence with NLS-Venus-GUS. I wonder whether this strategy could result in a reporter mRNA having long 5' UTR with spurious ATGs that will in turn affect its expression. Please comment.*

We did examine the untranslated regions of the fusion transcripts and have now annotated Figure S1 to indicate the ATGs that are not in introns and are upstream of the NVG reporter gene as well as the ATGs that are present upstream of the *MIR*  hairpin coding region that were intentionally omitted during cloning of these constructs. The gene structures of *MIR172A* and *MIR172B* have been annotated by TAIR and the transcriptional start sites for *MIR172C* and *MIR172E* were defined by Xie et al. (2005) Plant Physiology. In addition, we have now used publicly available RNA-Seq data to estimate the regions of *MIR172C-E* that are transcribed.

Although there are potential alternative ATGs that could be used to initiate translation of the NVG reporter, our RT-qPCR data (Fig 1A, Fig 4D) closely follow the expression patterns of our reporter constructs. Furthermore, the intense expression of most of these reporters does not suggest that these upstream ATGs significantly reduce the reliable translation of the NVG reporter. There are also limited alternative strategies in the construction of these fusions without omitting potential regulatory elements that may have large effects on the expression pattern.

5. Figures have low resolution. Figure 3C, the phenotype indicated by the arrow is not clear.

We believe that the poor resolution of the figures mentioned by the reviewer is due to the PDF conversion compression. High-resolution images of the required resolution will be provided for publication. The arrow in Figure 3C indicates the bolting of mutant plants from axillary meristems rather than the primary meristem, as mentioned in the legend.

6. "*Furthermore, The TLN and DTB of mir172ab", there is a typo ", The".*

We edited this typo that was also pointed out by reviewer 2, point 2.

7. *"…transition at the SAM, and initiated flowering from axillary branches, although most of these plants did eventually flower from the SAM as well (Fig 13A-C)." - there is a typo Fig S12A-C.”*

Thanks, we corrected that on page 23 to refer to Fig 7B.

8. "These results indicate that transcriptional activation of FUL and MIR172 by SPL15 may be essential for flowering of the SAM in some plants" -- typo FUL/MIR172 should be in italics.

Thanks, we edited that on page 24.

We would like to thank the reviewers for their constructive suggestions to improve the manuscript.

We hope that our revised version is now suitable for publication, and look forward to your decision.

Sincerely,

Dr George Coupland.
