# Supplementary material for: Systematic analyses of the MIR172 family members of Arabidopsis define their distinct roles in regulation of APETALA2 during floral transition
Source: PLoS Biol. 2021 Feb 2;19(2):e3001043. doi: 10.1371/journal.pbio.3001043 (PMC7853530; doi:10.1371/journal.pbio.3001043)
Supplement: Attachment — Submitted filename: Rebuttal letter second revised headed.docx [file pbio.3001043.s021.docx]

Dr Alvarez-Garcia,

Plant Developmental

Biology

Prof. Dr George Coupland

Tel.: +49-(0) 221-5062-205

Fax: +49-(0) 221-5062-207

coupland@mpipz.mpg.de

Cologne, 5^th^ December 2020

Editor,

*PLoS Biology*.

Dear Ines,

Thank you for your Email of 24^th^ November 2020 informing us that our manuscript "Analysis of the *MIR172* family defines transcriptional and post-transcriptional mechanisms that coordinately regulate APETALA2 to control floral transition of Arabidopsis" (PBIOLOGY-D-20-01959R1) had satisfied the requirements of the editorial board. We were very pleased to hear that our manuscript is close to acceptance. We are now resubmitting the second revised version that incorporates the new title as we subsequently discussed by Email, and includes the formatting changes and extra information requested by Jessica Hyde in her Email of 27^th^ November. We have considered all of the issues mentioned in the PLoS Formatting Requests check list and used the PACE figure-checking tool to proof our figures.

We hope that our manuscript can now proceed to acceptance and publication.

Yours sincerely,

Dr George Coupland
